# Comparative chloroplast genome analysis of *Polygala* species: insights into evolution, phylogeny, and DNA barcoding

**DOI:** 10.1007/s00425-026-04971-7

**Published:** 2026-03-17

**Authors:** İlayda Gülmez, Ali A. Dönmez, Zübeyde Uğurlu Aydın

**Affiliations:** Molecular Plant Systematic Laboratory (MOBIS), Department of Biology, Faculty of Science, Hacettepe University, 06800 Ankara, Türkiye

**Keywords:** Comparative analysis, DNA barcoding, Plastome, Phylogeny, *Polygalaceae*

## Abstract

*Polygala* species are rich in saponin molecules and are traditionally used for medical purposes. Research on the chloroplast (cp) genome of *Polygala* is crucial to understanding its phylogenetics, biogeography, and species identification. In this study, the tetraploid *Polygala vulgaris* chloroplast genome was reported for the first time via various assembly and annotation tools. By combining these new data with the published cp genomes of 12 *Polygala* taxa from NCBI, we conducted a comparative analysis of nucleotide diversity, repeat sequences, genome synteny, phylogenetic relationships and DNA barcoding. The final assembled chloroplast genome sizes for the studied *Polygala* taxa ranged from 165,246 bp to 168,779 bp, with a total of 174 genes annotated, including 102 protein-coding genes, 63 transfer RNA genes, and 13 ribosomal RNA genes in *Polygala vulgaris*. A total of nine highly divergent genes were identified, including the CDS gene *rpl32* as well as the intergenic spacer regions *matK*-*trnK*, *trnL*-*trnF*, *trnH*-*psbA*, *trnQ*-*psbK*, *ndhI*-*ndhF*, *ndhF*-*rpl32*, *ccsA*-*ndhD*, and *ndhG*-*ndhI*. Phylogenetic relationships were reconstructed based on Bayesian inference and maximum likelihood analyses using ortholog-based and concatenated datasets derived from the identified hypervariable regions separately. The topological structure of the constructed phylogenetic tree showed biogeographic disjunction, and the most similar relative of *Polygala vulgaris* was one of the European taxa, *P. amerella*, based on high support values. The analysis of relative synonymous codon usage revealed that a codon usage pattern largely consistent with that observed across other *Polygala *species. A total of three types of repeat sequences, forward, palindromic, and reverse, were identified across the chloroplast genomes of the analyzed species. These findings provide a valuable reference for phylogenomic and evolution of *Polygala* taxa as well as to develope molecular markers for DNA barcoding.

**Graphical abstract:**

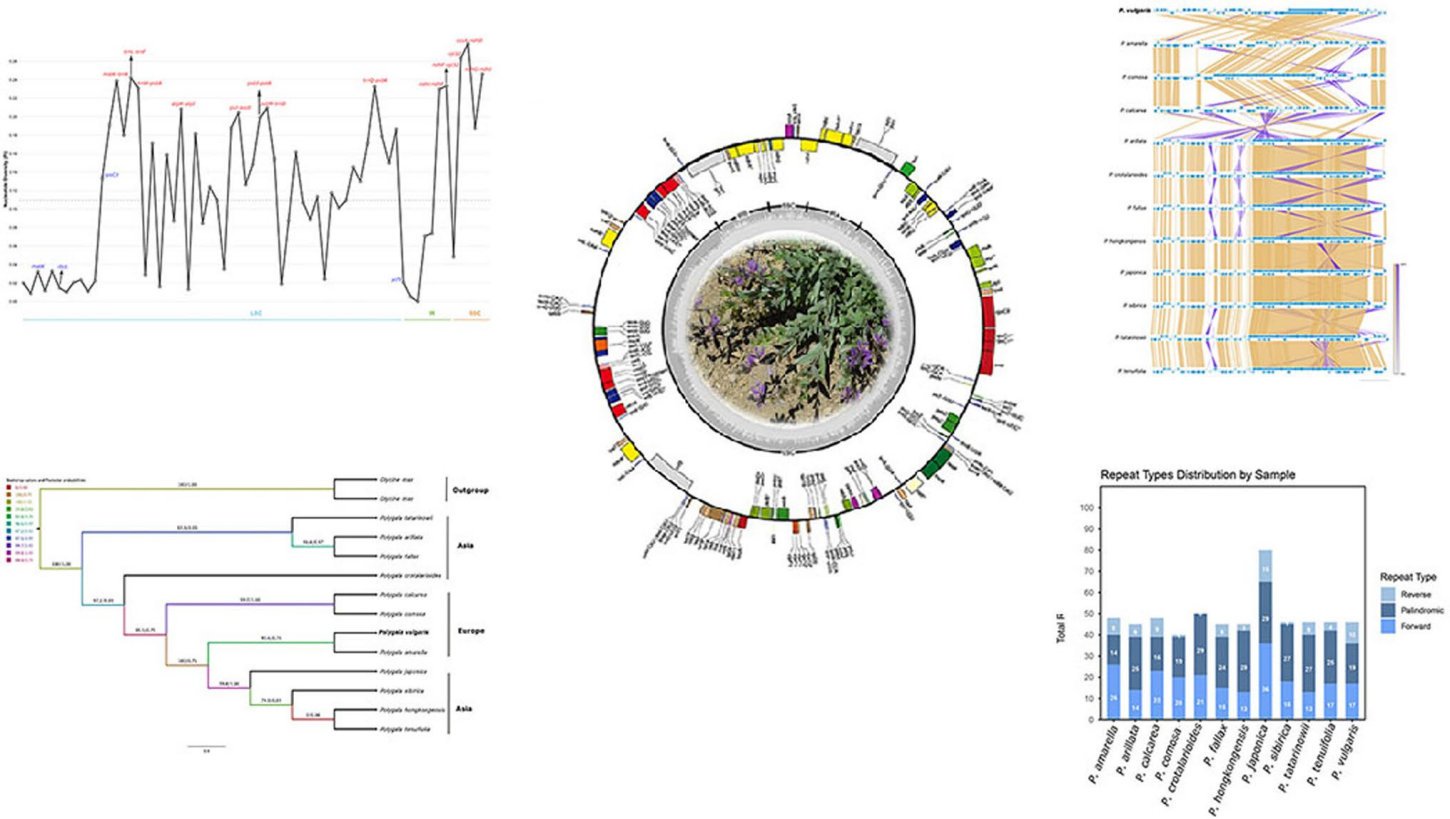

## Introduction

The genus *Polygala*, commonly known as milkwort, is a polyphyletic assemblage and belongs to the *Polygalaceae* family (Pastore et al. 2019). *Polygala* species are natively distributed worldwide, except in the Arctic and New Zealand (Eriksen and Persson 2007). Two well-supported clades of the genus are referred to as the New World Clade (NWC) and the Old World Clade (OWC) (Abbott 2009). The *Polygala* OWC is composed of more than 300 species of which 20 species are widespread in Türkiye including the recently described endemic species (Dönmez et al. 2024). Among them, *Polygala vulgaris* L. is widely distributed in Europe, and it also occurs in several locations in Türkiye. Cytogenetic data reveal that the number of *Polygala* chromosomes varies between species. However, *P. vulgaris* has been estimated to be diploid via flow cytometry (0.89 pg/2C, Horjales et al. 2003), and a tetraploid number of 2n = 68 has been reported in European and Turkish populations (Glendinning 1960; Şenova 2021).

The *Polygala* species includes several phytochemicals and is used in herbal medicines. Over the years, many phytochemicals have been successfully extracted from *Polygala* species (Çalış et al. 2022; 2023; Jing et al. 2024; Klein et al. 2012). The roots of *P. tenuifolia* (Willd.) Regel and *P. senega* L. are used for their expectorant, diuretic, and anti-inflammatory effects (Yoshikawa et al. 1995; 1996). Additionally, several studies have shown that *P. vulgaris* extracts have therapeutic benefits for neurodegenerative diseases (Çalış et al. 2023; Dall'Acqua et al. 2002).

The chloroplast (cp) is a dynamic photosynthetic organelle in green plant cells, and it is essential for metabolic activities such as photosynthesis and plant biosynthesis (Daniell et al. 2016; Neuhaus and Emes 2000; Serrano et al. 2016). The chloroplast genome represents relatively independent evolution, evolving slower than the nuclear genome but faster than the mitochondrial genome does, and it exhibits maternal inheritance. Compared with the nuclear genome, its genome is shorter and has highly conserved gene content and structure (Wicke et al. 2011). However, low rates of several chromosomal rearrangements, such as mutations, duplications, insertions/deletions, and inversions, have been reported (Amiryousef et al. 2018; Chung et al. 2006; Ghimiray and Sharma 2014). The typical angiosperm chloroplast genome is mostly circular in shape and has a quadripartite structure composed of two large copies of inverted repeats (IRa and IRb), which separate the small single-copy (SSC) and large single-copy (LSC) regions (Chung et al. 2006; Guzmán-Díaz et al. 2022; Liu et al. 2019). The chloroplast genome is between 72 and 220 kb in size and encodes 110–130 genes, with approximately eighty protein-coding genes, four rRNA genes, and thirty tRNA genes (Daniell et al. 2016).

The chloroplast genome plays an important role in plant molecular evolution and phylogenetic studies (Guzmán-Díaz et al. 2022), and it offers valuable genetic resources for complex phylogenetic relationships in angiosperms (Graham and Olmstead 2000) and polyploidization events in cultivated plants (Gornicki et al. 2014). Plastome analysis has been applied to obtain better results in the study of comparative genetic and phylogenetic relationships of polyploid plants, such as *Fragaria *L. Song et al. 2023a, b), and medicinal plants, such as *Nepeta* L. (Chen et al. 2023), *Thymus* L., and *Calendula* L. (Zhang et al. 2024), among others. Polyploid cytotypes are of particular evolutionary interest in plastid genome research because, although chloroplasts are typically maternally inherited in angiosperms, polyploidization can indirectly influence plastome evolution through cytonuclear interactions, genome dosage effects, and population-level genetic divergence (Gornicki et al. 2014; Soltis et al. 2015; 2014; Xiong et al. 2023). While the overall structure and gene content of chloroplast genomes are generally conserved across different ploidy levels, subtle differences in genome size, repeat content, mutation rates, and sequence variation between diploid and polyploid lineages have been reported in several plant groups (Li et al. 2025; Song et al. 2023a, b; 2025; Zhang et al. 2024). Moreover, polyploid lineages often exhibit broader ecological tolerance and geographic distributions, which may be reflected in plastid genome variation at the intraspecific level (Peer et al. 2017). Therefore, investigating the chloroplast genome of a tetraploid cytotype of *P. vulgaris* provides an opportunity to assess whether polyploidization is associated with detectable plastome-level variation and contributes to the evolutionary and phylogenetic interpretation of the species.

DNA barcoding, which uses short and standardized DNA fragments, was proposed as a quick and alternative identification method (Hebert et al. 2003). This genetic approach has been applied to many different taxa, and its ability to discriminate among species has been discussed (Bi et al. 2018; Chen et al. 2022; Hollingsworth et al. 2011; Uğurlu Aydın et al. 2021). Indeed, DNA barcoding have been widely used for medicinally important commercial plants to avoid misidentifications on the basis of morphological data (Chen et al. 2023). Therefore, Jeong et al. (2025) developed hot spot markers to accurate identification of two herbal medicine, *P. tenuifolia* and *P. sibirica* L. It is crucial to establish a rapid and accurate molecular method for other medical plants including *P. vulgaris*.

Currently, compared with the universal barcode region obtained from Sanger sequencing, the complete chloroplast genome sequence provides a powerful tool for species identification (Chen et al. 2020; Wu 2021b).

Owing to high-throughput sequencing technologies, vast amounts of DNA data, including both nuclear and organelle genomes, have been generated in land plants, including *Polygala* taxa. Recently, comparative chloroplast (hereafter cp) genome analyses of several *Polygala* species have revealed nucleotide variations and genomic structures crucial for phylogenetic relationships and species identification (Jeong et al. 2025; Wang 2023).

In the present work, the cp genome of a tetraploid *P. vulgaris* individual’s was analyzed for the first time via short-read DNA sequences. On the basis of cp genome sequences, we determined the genome structure, gene content, tandem repeats, and phylogenetic relationships among *Polygala* taxa. Moreover, we aimed to identify the most variable regions of the cp genome as potential DNA barcode markers and to test discrimination power of plastome-derived hot spot regions.

## Materials and methods

### Plant materials and DNA extraction

Fresh young leaves of *P. vulgaris* collected from Erzurum, Türkiye, were subsequently dried with silica gel. Voucher samples were stored at the HUB herbarium of Hacettepe University (*A.A. Dönmez* 20628). The samples’ DNA was extracted with a DNeasy Plant Mini Kit (Qiagen Co., Germany). The quality and quantity of the genomic DNA were checked on a Qubit fluorometer. Three individuals of *P. vulgaris* were initially sequenced and all chloroplast genome assembly and downstream analyses were conducted using a representative individual (A.A. Dönmez 20628_1), which is therefore the only specimen reported in the Results.

### Genome sequencing, assembly, and annotation

The whole-genome sequencing was carried out via the TruSeq Nano DNA Kit (Illumina, San Diego, CA, USA). Approximately 4 Gb of raw data were generated for *P. vulgaris* (A.A. Dönmez 20628_1) using 150 bp paired-end whole-genome sequencing. Based on an estimated nuclear genome size of ~ 1.82 Gb (Şenova 2021), this corresponds to an average whole-genome sequencing depth of approximately 2.2 × **.**

According to the dataset, eleven *Polygala* chloroplast genomes were retrieved from NCBI as complete assembled plastomes, whereas chloroplast genomes of two additional species were assembled in this study from publicly available whole-genome sequencing (WGS) datasets (Table 1). GenBank accessions correspond to complete plastome sequences downloaded directly from NCBI, while SRA accessions refer to raw WGS data used for de novo chloroplast genome assembly using GetOrganelle (Jin et al. 2020). Low-quality reads and adapters were filtered from the raw data via Trimmomatic (Bolger et al. 2014) with the default settings. The plastid assembly was performed from the clean reads via a reference-based method using the program GetOrganelle, with *k*-mers of 21, 45, 65, 85, and 105. Gene annotation of the cp genomes was performed via GeSeq (https://chlorobox.mpimp-golm.mpg.de/geseq.html). Protein-coding sequences (CDSs) were extracted manually from Geneious (Kearse et al. 2012) and aligned with MAFFT (Katoh et al. 2002). For orthology-based comparisons among taxa, annotated protein-coding genes were translated, and orthogroups were inferred using OrthoFinder (Emms and Kelly 2019). Where necessary, protein sets were standardized using AUGUSTUS-based gene prediction to ensure consistent input for orthogroup inference (Stanke et al. 2006). tRNA genes were identified via tRNAscan-SE 1.21 (Schattner et al. 2005). All *Polygala* taxa cp maps were obtained via OGDRAW (https://chlorobox.mpimp-golm.mpg.de/OGDraw.html).

**Table 1:** Comparison of the chloroplast genome data in this study

Sample	NCBI accession (GenBank/SRA)	LSC (bp)	LSC GC (%)	SSC (bp)	SSC GC (%)	IRA (bp)	IRB (bp)	IRs GC (%)	Sequence length (bp)	Total GC (%)	Protein coding gene (CDS)	tRNA	rRNA
***P. vulgaris***	SRR32576552	83,543	37,9	7994	29,5	36,736	36,736	31,9	165,009	36,6	102	63	13
*P. comosa Schkuhr*	ERX13411377	87,438	35,0	7863	29,8	34,525	34,525	39,8	164,351	36,8	107	66	14
*P. amarella Crantz*	ERR5554716	133,648	37,9	7840	29,9	11,568	11,568	32,1	164,624	36,7	77	39	8
*P. calcarea F. W. Schultz*	ERR14043099	133,368	38,0	7873	29,8	11,547	11,547	32,1	164,335	36,8	77	39	8
*P. tenuifolia*	NC_050829	83,696	34,7	8044	29,4	36,840	36,840	39,7	165,42	36,7	100	66	14
*P. arillata Buch.-Ham ex D. Dıb*	SRX22362821	83,537	34,9	8210	30,1	36,600	36,600	39,8	164,947	36,8	103	66	10
*P. crotalarioides B. -Ham. ex DC.*	NC_060367	86,873	35,1	8246	29,2	34,574	34,575	39,8	164,268	36,8	102	67	14
*P. fallax Chodat*	NC_052911	83,352	35,0	8283	29,7	36,526	3656	39,9	164,687	36,9	100	66	14
*P. hongkongensis Hemsl. ex F.B.Forbes & Hemsl.*	NC_066219	83,707	34,8	8035	29,6	36,777	36,777	39,7	165,296	36,7	101	66	14
*P. japonica Houtt.*	NC_052912	88,128	34,9	8084	29,5	34,517	34,517	39,8	165,246	36,7	101	66	14
*P. sibirica*	NC_056970	83,668	34,8	8095	29,5	36,742	36,742	39,7	165,247	36,7	101	66	14
*P. tatarinowii Regel*	NC_086943	73,712	34,9	8213	29,0	43,427	43,427	39,1	168,779	36,8	111	67	14

The new sequence from this study is in boldface

### Analysis of repeats

We identified forward (F), reverse (R), and palindromic (P) repeat sequences via the online tool REPuter v1.0 (Kurtz et al. 2001). The analysis was conducted with conditions specifying a minimum repeat size of 8 base pairs and a maximum of 50 computed repeats. Dispersed repeat analysis performed using REPuter, tandem repeats and simple sequence repeats (SSRs) were automatically identified using the cpGAVAS annotation pipeline, which integrates the MISA (MIcroSAtellite identification tool; Thiel et al. 2003) algorithm. Mononucleotide, dinucleotide, trinucleotide, tetranucleotide, pentanucleotide, and hexanucleotide SSRs were detected using the default parameters of cpGAVAS.

### Comparison of the chloroplast genome

We extracted conserved CDSs and some gene regions via Geneious software. The nucleotide diversity (Pi) value between the *Polygala* cp genomes was subsequently calculated via DnaSP ver.6 (Librado and Rozas 2009) with a sliding window analysis. The step size was set to 50 bp, and the window length was set to 600 bp. Plots are generated with ggplot2 via R. Using pyGenomeViz (https://github.com/moshi4/pyGenomeViz), we created a synteny plot employing the MMseqs RBH mode and setting an identity threshold of 50%.

A comparative visual analysis of the boundaries of inverted repeat (IR) sequences was performed via IRscope Plus (https://irscope.shinyapps.io/IRplus/). Additionally, relative synonymous codon usage (RSCU) analysis of the chloroplast genome of *Polygala* taxa was performed via the codonW (https://galaxy.pasteur.fr/?form=codonw) online software.

### Phylogenetic reconstruction

A Bayesian inference (BI) phylogenetic tree was constructed via MrBayes v3.2.7 (Ronquist et al. 2012), which employs the Markov chain Monte Carlo (MCMC) method. Four chains (three heated and one cold) were run for 10,000,000 generations, with a sampling frequency of 1000 and a printing frequency of 10,000. A burn-in of 2500 samples (corresponding to 25% of the total) was applied to ensure convergence. A 50% majority-rule consensus tree was generated to summarize the posterior distribution of trees.

Second, a maximum likelihood (ML) tree was generated via IQ-TREE v2.2.0 (Nguyen et al. 2015) under the best-fit model automatically selected via ModelFinder (cpREV + R2). Support for the nodes was assessed via 1000 ultrafast bootstrap replicates. Finally, both the BI and ML trees were compared, and the final consensus tree was annotated to include both posterior probabilities (from MrBayes) and bootstrap support values (from IQ-TREE). FigTree v1.4.4 (https://tree.bio.ed.ac.uk/software/figtree/) was used to visualize and annotate the combined tree topology. Nine highly variable chloroplast regions, (*ccsA*–*ndhD*, *matK*–*trnK*, *ndhF*–*rpl32*, *ndhG*–*ndhI*, *ndhI*–*ndhF*, *rpl32*, *trnH*–*psbA*, *trnL*–*trnF*, and *trnQ*–*psbK*), were extracted from chloroplast genomes and aligned individually using Geneious Prime. Sequence alignments were inspected manually to ensure positional homology, particularly within intergenic spacer regions. To infer species-level phylogenetic relationships, the nine aligned barcode regions were concatenated into a single supermatrix using AMAS v1.0. (Borowiec 2016). Maximum likelihood phylogenetic inference based on the concatenated dataset was performed using IQ-TREE. Partitioned analyses were conducted by applying the partition scheme produced by AMAS, allowing each gene region to evolve under its own substitution model. The optimal substitution model for each partition was selected automatically using ModelFinder implemented in IQ-TREE. Branch support was assessed using 1000 ultrafast bootstrap replicates. The resulting phylogenetic tree was visualized using FigTree.

## Results

**Fig. 1:**
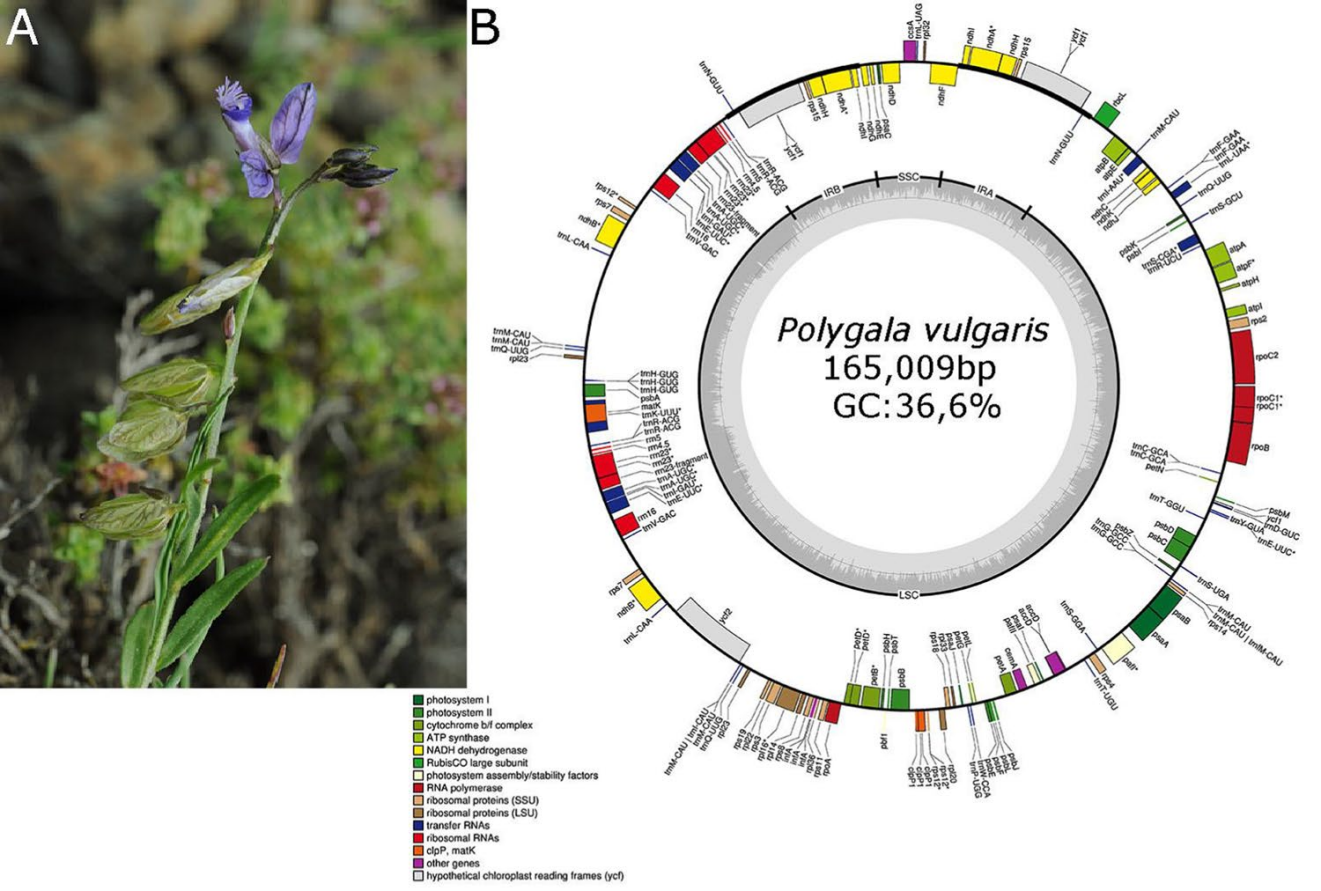
**A** General view of *Polygala vulgaris* flower and fruit. **B** Physical map of the *P. vulgaris* chloroplast genome. Colored boxes represent genes grouped by functional categories. The innermost grey circle indicates GC content (%)

We obtained approximately 4 Gb of trimmed paired-end reads. The chloroplast genome was then generated through de novo assembly. The completed circular genome length was 165,009 bp (Fig. 1). The tetraploid *P. vulgaris* cp genome has a typical quadripartite structure similar to that of angiosperm plastid genomes, consisting of an LSC (83,543 bp) and SSC (7,994 bp), separated by a pair of identical IRs (11,636 bp and 36,736 bp). The overall guanine‒cytosine (GC) content of the *P. vulgaris* cp genome is 36,6%, which is nearly identical to that of other *Polygala* cp genomes, although it is unevenly distributed within the cp genome. Moreover, the highest GC content was in the LSC region (37,9%), and the GC contents in the SSC and IR regions were 29,5% and 31,9%, respectively (Table 1).

The chloroplast genome of *P. vulgaris* is 165,009 bp in length and contains a total of 174 genes, including 102 protein-coding genes, 63 transfer RNA (tRNA) genes, and 13 ribosomal RNA (rRNA) genes. Among these genes, six protein-coding genes (*atpF*, *rpoC1*, *rpl16*, *rpl2*, *ndhA*, *and ndhB*) and seven tRNA genes (*trnK*-*UUU*, *trnL*-*UAA*, *trnV*-*UAC*, *trnI*-*GAU*, *trnA*-*UGC*, *trnE*-*UUC*, *and trnS-CGA*) contained one intron, whereas one protein-coding gene (*pafI*) contained two introns. In addition, *rps12* was identified as a trans-splicing gene (Fig. 1; Table 2).

**Table 2:** List of genes annotated in the chloroplast genomes of twelve *Polygala* species

Group of genes	Name of genes
Photosystem I	psaA, B, C, I, J, pafI**, pafII
Photosystem II	*psbA, B, C, D, E, F, H, I, J, K, L, M, T, Z, pbf1*
Cytochrome b6/f	*petA, B, D (× 2), G, L, N*
ATP synthase	atpA, B, E, F*, H, I
RubisCO large subunit	*rbcL*
NADH oxidoreductase	ndhA* (× 2), B*, C, D, E, F, G, H, I, J, K
Large subunit ribosomal proteins	rpl2* (× 2), 14, 16*, 20, 23, 32, 33, 36
Small subunit ribosomal proteins	rps2, 3, 4, 7, 8, 11, 12** (× 2), 14, 15, 18, 19
RNA polymerase	rpoA, B, C1* (× 2), C2
Unknown function protein coding gene	*ycf1 (× 2), ycf2*
Other genes	*accD (× 2), ccsA, cemA, clpP, infA (× 2), matK*
Ribosomal RNAs	rrn4.5, rrn5, rrn16, rrn23*
Transfer RNAs	trnA-UGC*(× 2), trnC-GCA, trnD-GUC, trnE-UUC*, trnF-GAA, trnM-CAU, trnG-GCC, trnH-GUG, trnl-CAU, trnl-GAU*, trnK-UUU*, trnL-CAA, trnL-UAA*, trnL-UAG, trnM-CAU(× 2), trnN-GUU(× 2), trnP-UGG, trnQ-UUG, trnR-ACG, trnR-UCU, trnS-CGA*, trnS-GCU, trnS-GGA, trnS-UGA, trnT-GGU, trnT-UGU, trnV-GAC, trnV-UAC*, trnW-CCA, trnY-GUA

The genes marked with the sign are the genes with one (*) or two (**) introns and duplicated genes (× 2)

### Contraction and expansion of inverted repeats

**Fig. 2:**
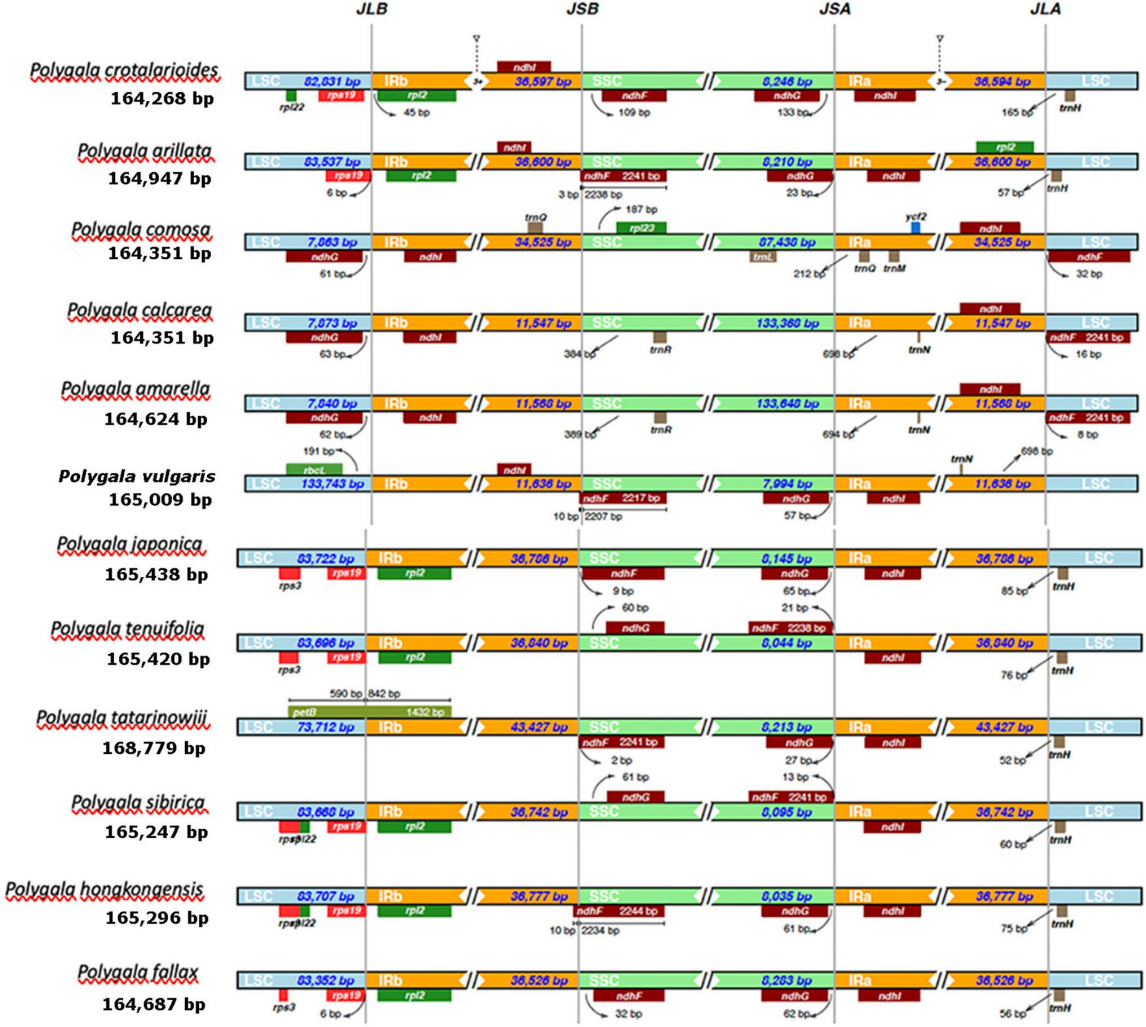
A comparison of the distance between adjacent genes and junctions of the small single-copy (SSC), large single-copy (LSC), and two inverted repeat (IR) regions in the *Polygala* species

The expansion and contraction of IR regions in the chloroplast genome are highly important in genome evolution. The IR regions are well conserved in cp genomes, but the IR/SC boundary regions differ among plant species. In this study, the LSC, IRa, IRb, and SSC junction positions and the distributions of the IR and SC border regions in the chloroplast genomes of twelve *Polygala* species were compared (Fig. 2).

In *P. vulgaris,* the IRb/SSC (JSB) junction was located within the *ndhH* gene, extending 2,417 bp into the SSC region, whereas the IRa/SSC (JSA) junction was positioned within *ycf1*, overlapping by 7,994 bp. Compared with other *Polygala* species, *P. vulgaris* presented a relatively longer LSC region (83,743 bp), but the IR regions (11,838 bp) were shorter than those observed in most congeners. These differences suggest lineage-specific expansions and contractions of IR boundaries in *P. vulgaris*, whereas the overall IR/SC organization remained conserved across the genus.

**Fig. 3:**
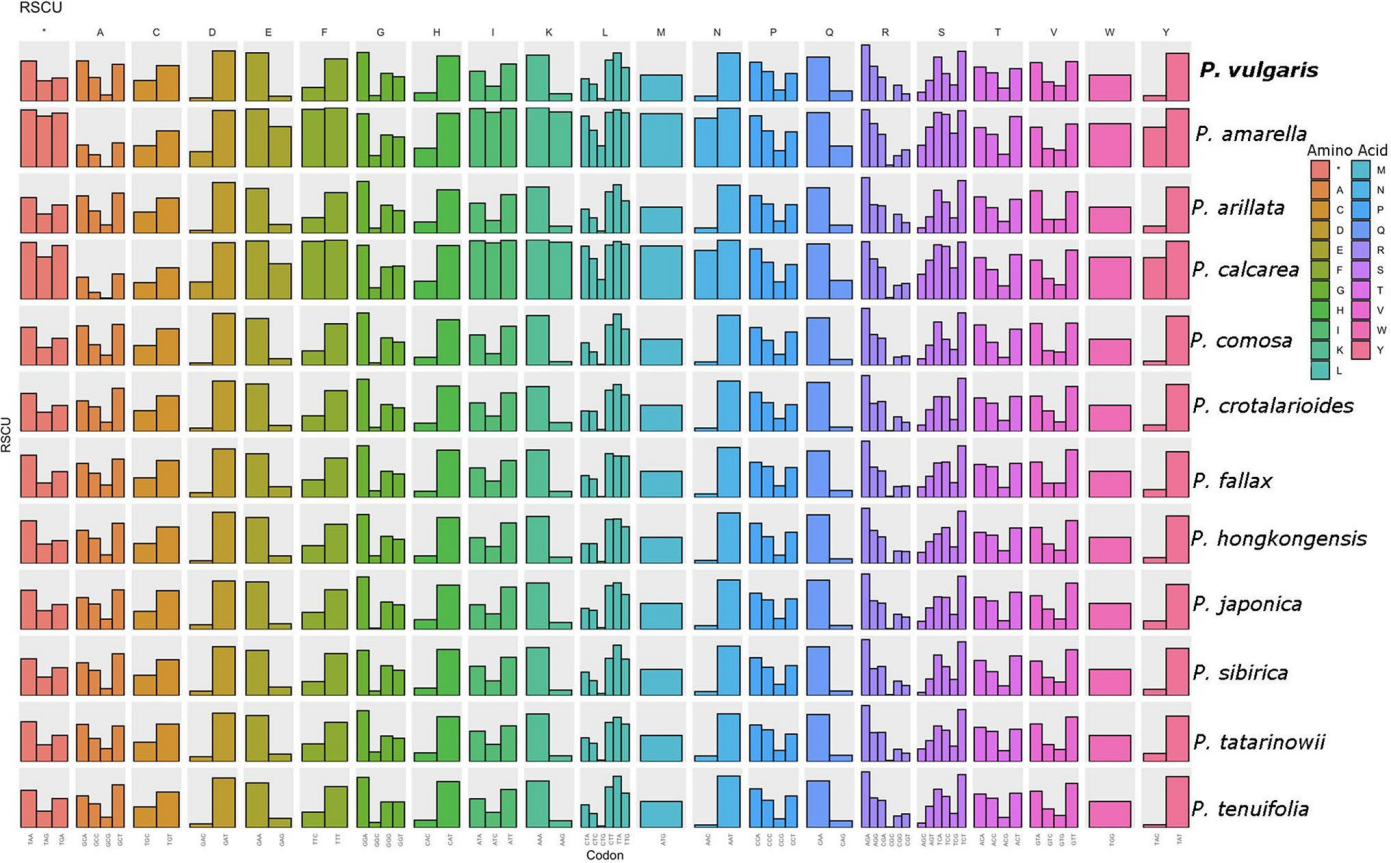
Relative synonymous codon usage (RSCU) in all *Polygala* taxa for this study

The relative synonymous codon usage (RSCU) analysis conducted in this study revealed that *P. vulgaris* presented a codon usage pattern largely consistent with that of the other *Polygala* species examined (Fig. 3). While overall codon preferences are conserved across the genus, certain codons display species-specific variations in usage frequency. Our findings suggest that codon usage patterns in *Polygala* chloroplast genomes are shaped by both conserved evolutionary constraints and species-specific selective forces.

RSCU analysis revealed similar codon usage patterns among all the examined *Polygala* taxa (Fig. 3). Although overall trends were largely conserved, certain codons showed clear preferences, reflecting species-specific codon bias. Our own sample displayed codon usage patterns consistent with those of other *Polygala* species, suggesting a conserved evolutionary codon preference within the genus.

**Fig. 4:**
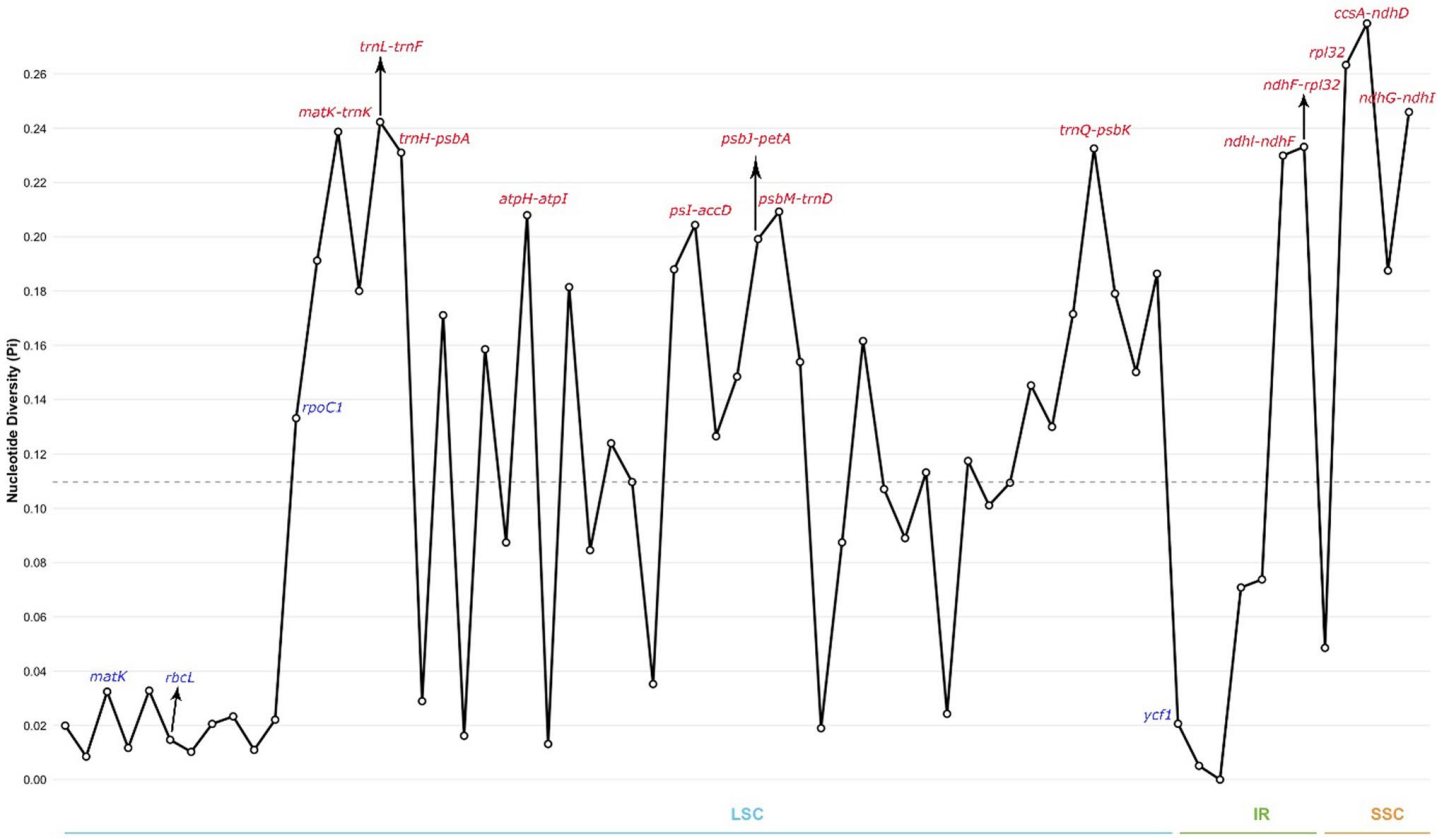
Nucleotide diversity (Pi) analysis of chloroplast genomes in *Polygala* species. The dashed line represents the mean Pi value (0.10970328), Window length: 600 bp, step size 50 bp

The nucleotide diversity (Pi value) within the sliding window of 600 bp was calculated to clarify hotspot regions in the genome (Fig. 4). The Pi value in the windows varied from 0.00509 to 0.27864, with a mean of 0.10970. Nine highly variable regions (Pi > 0.22) were identified in the *Polygala* genome. Among these regions, *matK*-*trnK*, *trnL-trnF*, *trnH-psbA*, and *trnQ*-*psbK* were located in the LSC region, and *ndhI*-*ndhF* and *ndhF*-*rpl32* were located in the IR region. The remaining three regions, *rpl32*, *ccsA*-*ndhD*, and *ndhG*-*ndhI,* were nested in the SSC region. Furthermore, the *ccsA*-*ndhD* region was the most highly divergent region (Pi value = 0.27864). Some of the universal DNA barcode markers, such as *matK* and *rbcL*, presented lower Pi values, whereas *trnL*-*trnF* and *trnH*-*psbA* showed greater nucleotide variation.

**Fig. 5:**
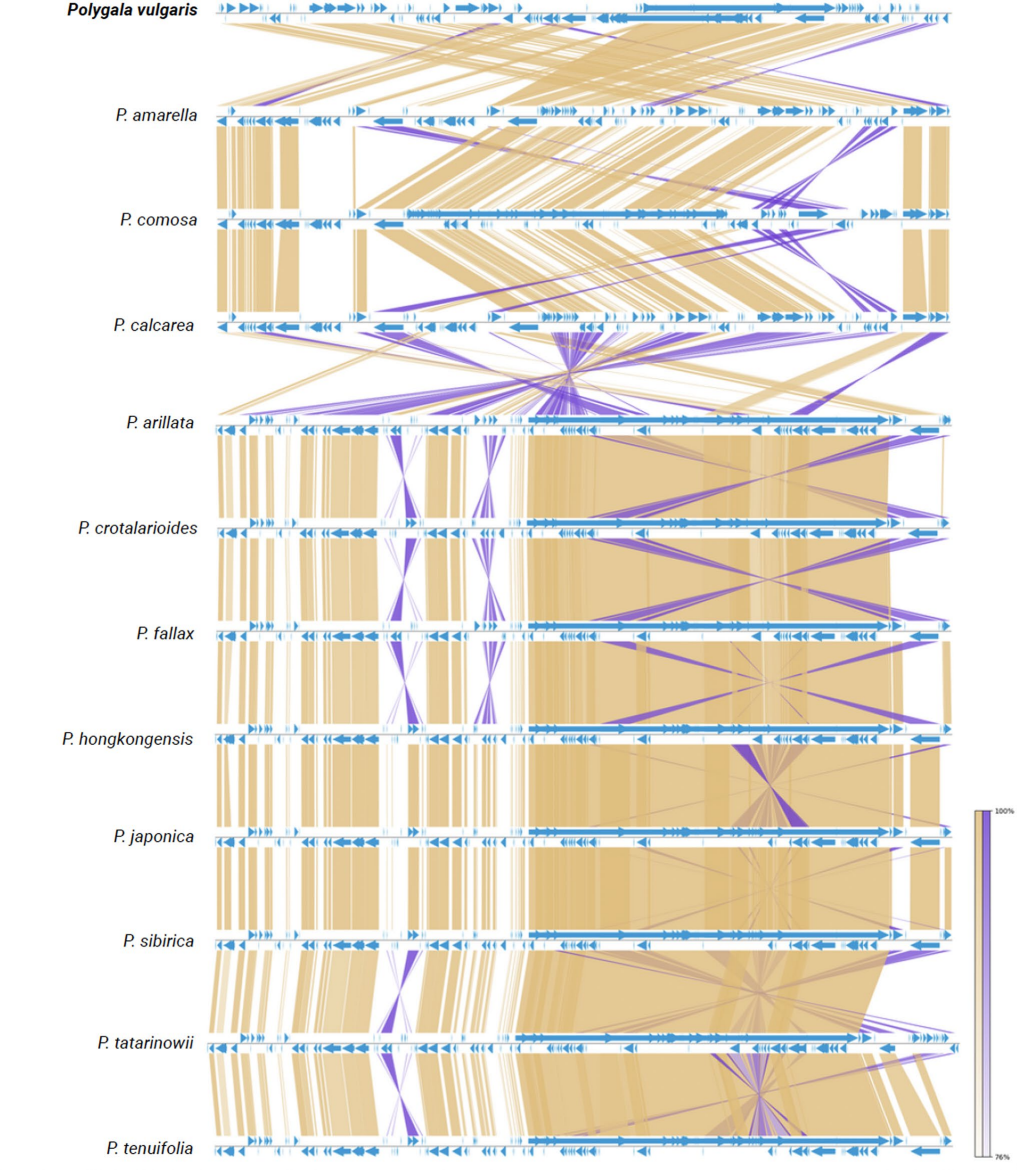
Synteny plot of *Polygala vulgaris* and eleven other plastomes of some *Polygala* taxa. The synteny plot shows normal links in yellow, inverted links in purple, and gene features in blue

The analysis revealed extensive collinearity across the plastomes, as indicated by the numerous yellow links, which represent conserved gene blocks (Fig. 5). Despite the overall conservation, several inverted regions (purple links) were detected, particularly in comparisons with *P. calcarea*, *P. arillata*, and *P. sibirica*, suggesting lineage-specific rearrangements. The gene features (blue) showed a largely consistent distribution across all species, reflecting the overall stability of plastid genome organization in the genus. Taken together, these results indicate that *Polygala* plastomes are highly syntenic, with only localized inversion events contributing to structural variation among species.

**Fig. 6:**
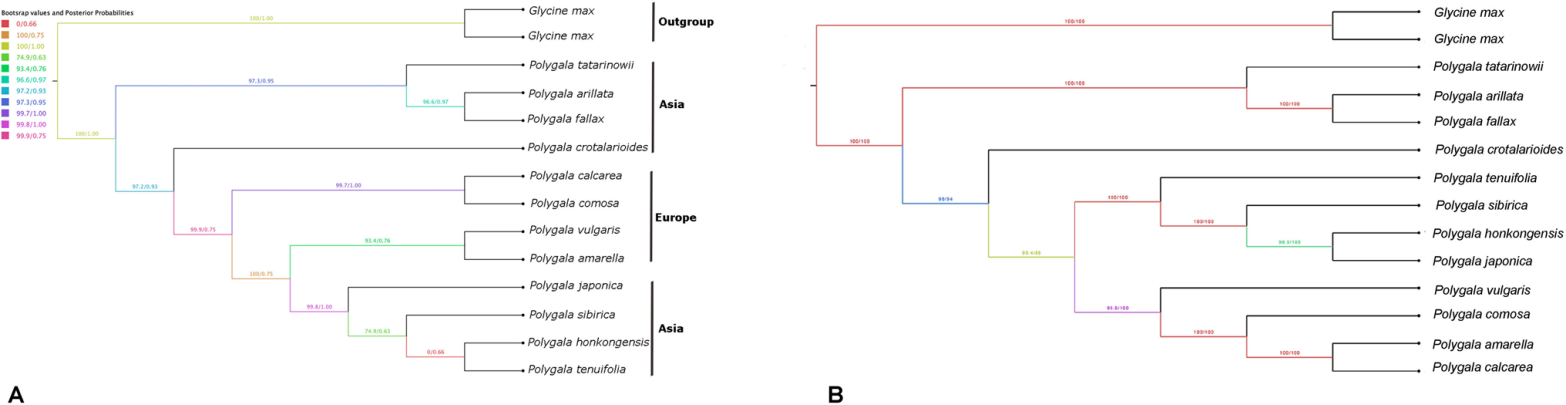
Phylogenetic trees of studied *Polygala* taxa. **A** Phylogenetic tree based on orthologous genes of chloroplast genome. **B** Phylogenetic tree based on hypervariable regions of chloroplast genome. The number above each node represents the bootstrap values (left) and posterior probabilities (right)

In order to create robust phylogeny of *Polygala* taxa, a phylogenetic tree was established using orthologous genes of the chloroplast genome (Fig. 6A). Moreover, discrimination power of hypervariable regions, suggested in this study, were tested based on ML and BI analyses (Fig. 6B). Two *Glycine* species were nested with high support values and served as outgroups. The tree topology of ML and BI analyses were identical, and the analyses were merged into a one tree.

Phylogenetic tree based on orthologues revealed that tetraploid *P. vulgaris* clustered with other morphologically similar diploid European species, *P. amarella* (PP = 0.76, MLBS = 93.4%). Clustering revealed that *P. vulgaris* was distinct from its sister groups, *P. japonica*, *P. sibirica, P. hongkongensis*, and *P. tenuifolia* (PP = 0.75, MLBS = 100%). Two other European taxa, *P. calcarea* and *P. comosa,* nested as sister clades to *P. vulgaris* and *P. amarella* (PP = 0.75, MLBS = 99.9%).

Based on the nucleotide diversity analysis, nine highly variable chloroplast regions were aligned and concatenated into a single dataset, which comprised all sampled taxa with consistent sequence representation across regions and showed no evidence of chimeric or duplicated sequences. Maximum likelihood and Bayesian analysis of the concatenated barcode dataset produced a well-resolved topology with strong support values for most internal nodes. The resulting tree topology was largely congruent with the phylogenomic tree inferred from whole chloroplast genes. Tetraploid *P. vulgaris* clustered with the European taxa *P. amarella*, *P. calcarea*, and *P. comosa*, forming a strongly supported clade. Asian taxa (*P. tenuifolia*, *P. sibirica*, *P. japonica*, and *P. hongkongensis*) were recovered as a separate lineage with high bootstrap support.

**Fig. 7:**
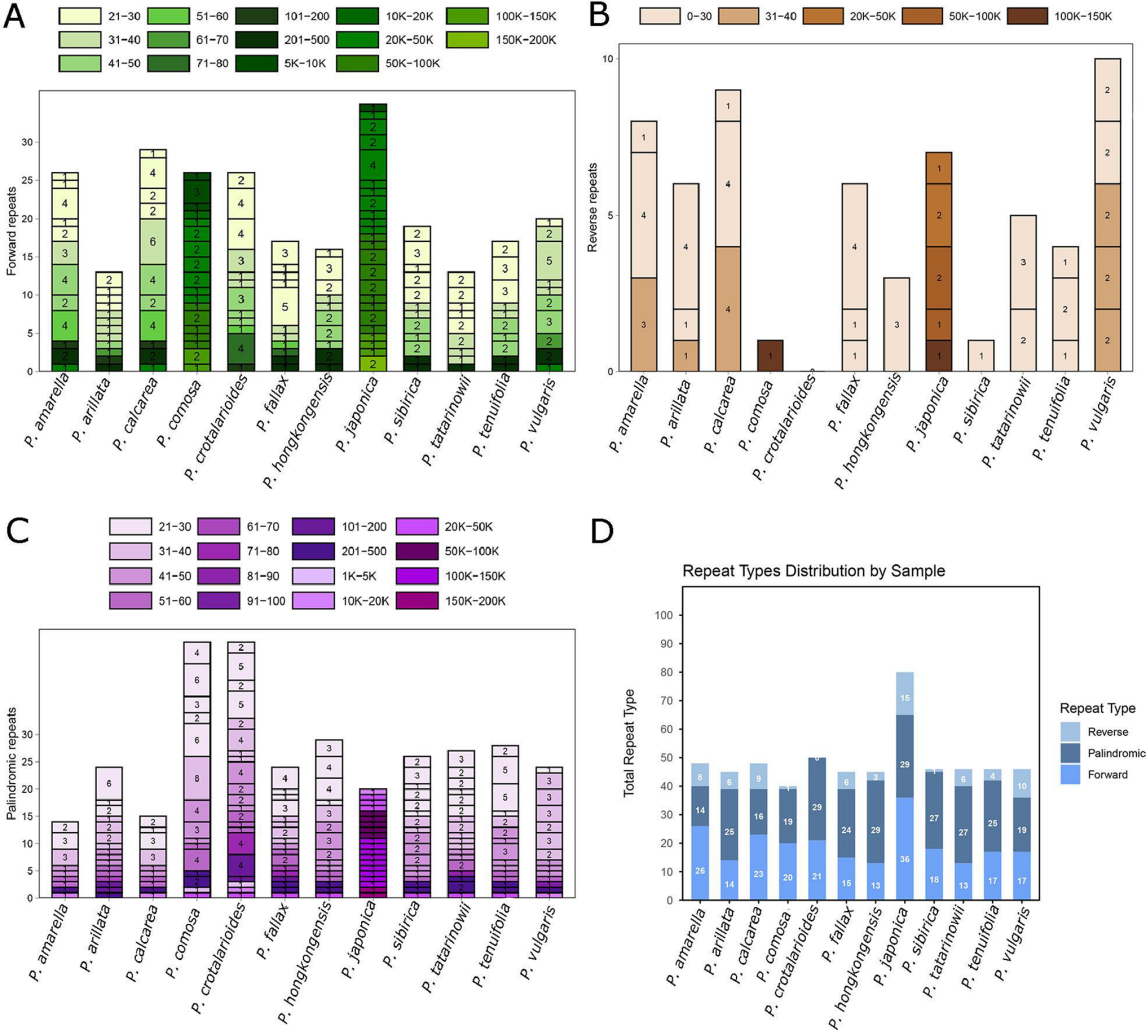
Distribution of repeat sequences in the plastid genomes of *Polygala* species. **A** The number and size categories of forward repeats are shown for each species. **B** The number and size categories of reverse repeats detected in the plastomes. **C** The number and size categories of palindromic repeats across species. **D** Comparative summary of the total number of repeat types (forward, reverse, palindromic) identified in each species

**Fig. 8:**
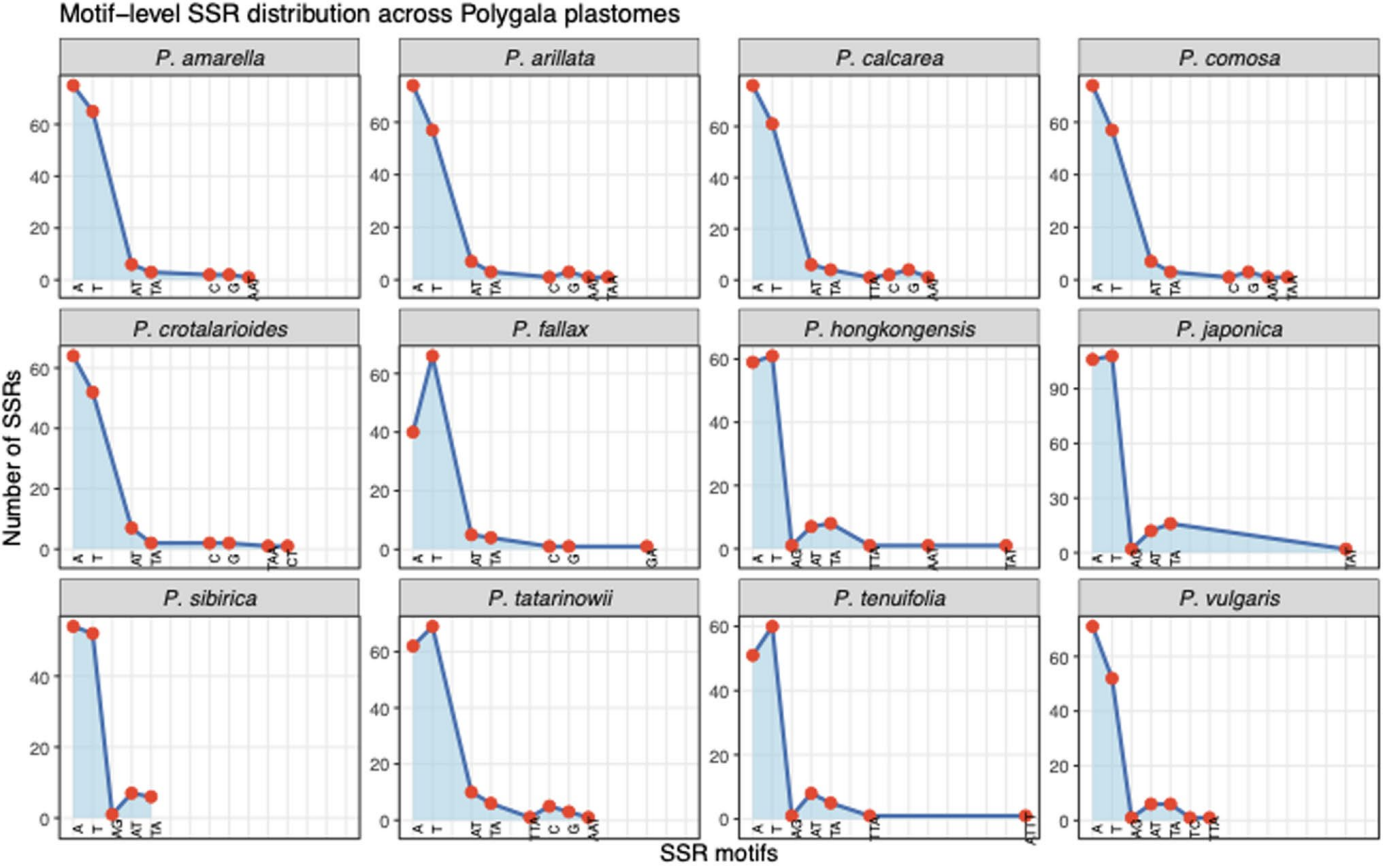
Motif-level distribution of simple sequence repeats (SSRs) across *Polygala* plastomes.

A total of three types of repeat sequences, forward, palindromic, and reverse, were identified across the chloroplast genomes of the analyzed species (Fig. 7). The total number of repeats varied among the species, ranging from 40 in *P. comosa* to a maximum of 80 in *P. japonica*. Among the repeat types, forward repeats were the most abundant in the majority of the species. The highest number of forward repeats (36) was observed in *P. japonica*, followed by *P. amarella* (26) and *P. calcarea* (23). Palindromic repeats were also commonly found, with the highest counts in *P. japonica* (29), *P. hongkongensis* (29) and *P. crotalarioides* (29). Reverse repeats were relatively less common across all the species, with a maximum of 15 detected in *P. japonica*. Also, a total of 118 to 157 simple sequence repeats (SSRs) were identified across the analyzed *Polygala* plastomes using the cpGAVAS–MISA pipeline (Fig. 8). Mononucleotide repeats were the most abundant class in all species, ranging from 106 in *P. sibirica* to 143 in *P. calcarea*, and accounting for the vast majority of detected SSRs. Dinucleotide repeats were represented by 9 to 16 motifs depending on the species, whereas trinucleotide repeats were rare and varied between 0 and 3. Tetra-, penta-, and hexanucleotide SSRs were largely absent from most plastomes; only *P. tenuifolia* harbored a single tetranucleotide repeat. Overall, A/T mononucleotide motifs dominated all plastomes, followed by a smaller proportion of AT/TA dinucleotide repeats.

Compared with the other taxa, *P. japonica* presented the highest repeat content overall, which may suggest a greater degree of genome rearrangement or structural variation in its chloroplast genome. In contrast, species such as *P. comosa* presented the lowest number of repeats, indicating a more conserved chloroplast genome architecture.

## Discussion

In this study, we utilized next-generation methods to sequence, assemble, and characterize the first complete plastid genome of *P. vulgaris*. The results revealed that the cp genome size of the studied *Polygala* species ranged from 164,268 in *P. crotalarioides* to 168,779 bp in *P. tatarinowii* (Table 1). Compared with the cp genomes of other *Polygala* species (Jeong et al. 2025; Lee et al. 2020; Wang 2023), the cp genome of *P. vulgaris* shares typical features with a typical quadripartite structure, with the LSC and SSC regions separated by two IRs.

Genome size variation and gene order are relatively conserved within terrestrial plants. Significant plastome size variation and structural rearrangements have been reported in the closely related family *Fabaceae*, primarily driven by expansion, contraction, or loss of the inverted repeat (IR) region (Li et al. 2025; Perry and Wolfe 2002; 2025). Similar patterns of IR loss have also been documented in other angiosperm lineages, including genera within *Arecaceae* (e.g., *Phoenix* and *Cocos*; Barrett et al. 2016), *Fabaceae* (e.g., *Medicago*, *Pisum*, and *Trifolium*; Choi et al. 2019), and *Passifloraceae* (e.g., *Passiflora*; Cauz-Santos et al. 2020). In *Fabaceae*, repeated loss and secondary regain of the IR region have been linked to extensive plastome rearrangements, accelerated substitution rates, and instability of genes located near IR boundaries, particularly members of the *ndh* and *ycf* genes. In contrast, all *Polygala* plastomes analyzed in this study exhibit a typical quadripartite structure with two intact IR regions, and no evidence of IR contraction, expansion, or loss was detected. Genes that are frequently affected by IR boundary shifts in other angiosperms, such as *ndhF*, *ycf1*, and *ycf2*, showed conserved positions and intact coding sequences across all examined *Polygala* species. The maintenance of IR structure in *Polygala* therefore indicates a high level of plastome stability. It suggests that the evolutionary dynamics observed in IR-labile families such as *Fabaceae* are not mirrored in *Polygalaceae*. Because gain and loss of the IR region are considered evolutionarily significant processes influencing plastome rearrangements, gene dosage effects, and substitution rates, the conserved IR architecture observed in *Polygala* provides further evidence for the structural stability of the plastid genome in this genus.

Compared with the ancestral organization of angiosperms, the *Polygalaceae* plastid genome has typical rearrangements (Ruhlman and Jansen 2014). The plastome of autotroph *Polygala* species is significantly larger than that of nonphotosynthetic species of *Epirixanthes* within the family (Petersen et al. 2019). *P. vulgaris*, an autotroph member of *Polygalaceae* included here, encoded a total of 174 genes, including 102 protein-coding genes (PCGs), 63 transfer RNA (tRNA) genes, and 13 ribosomal RNA (rRNA) genes (Table 1).

The guanine and cytosine (GC) contents are important for understanding genetic and species diversity (Liu et al. 2023; Singh et al. 2016; Smarda et al. 2014). The cpDNA GC content of *P. vulgaris* is highly similar to that of other studied *Polygala* taxa, in that it is approximately 36%; the GC content in the LSC, SSC, and IR regions varies between 29 and 39%. In addition, the GC content of IRs (between IRs 31 and 39%) is greater than that of LSCs (between 34 and 38%) and SSC regions (29–30%), which is in agreement with the results of previously reported studies of different genera (Hu et al 2023; Ma et al. 2021; Yan et al; 2023). This phenomenon might be attributed to the rRNA sequences with high GC contents being located in the IR regions.

The Consortium for the Barcode of Life (CBOL) (2009) has recommended the use of *matK* + *rbcL* as the core barcode based on primer universality, PCR amplification success and species discrimination power. Additionally, other barcode regions, such as *rpoC1*, *rpoB*, *psbA*–*trnH*, *psbK*–*psbI*, and *atpF*–*atpH*, *trnL*-*trnF*, and *ITS*, have been suggested to distinguish closely or distantly related taxonomic groups of land plants (Cowan et al. 2006; Hollingsworth et al. 2011; Kress et al. 2005; Uğurlu Aydın 2021). These hypervariable regions can serve as potential mini-barcodes for biodiversity assessment and conservation especially in economical important or high-risk plant groups (Kress et al. 2015). Among chloroplast DNA barcoding markers, only the *matK*-*trnK* intergenic spacer region was used to construct a phylogenetic tree (Pastore et al. 2019), and the remaining hotspot markers have not been used for efficient species identification in *Polygala*. Nucleotide variation analyses revealed that two potential barcode regions, *trnL‒trnF* and *trnH‒psbA,* can have enough discrimination power for DNA barcoding. Among them, only the *trnL‒trnF* region was employed as a plant DNA barcode locus for *Polygala* taxa (Aygören Uluer 2021). Otherwise, *ycf1* was screened for species identification and suggested as one of the most promising DNA barcoding regions for land plants on the basis of its discrimination power (Dong et al. 2015), but the region had such low variation (Pi > 0.2) to be selected as a DNA barcoding marker for *Polygala*.

In this study, all hot spot regions were employed further supports the robustness of the phylogenetic relationships inferred from whole plastome data. The concatenated barcode-based phylogeny demonstrated that the selected hypervariable chloroplast regions provide sufficient phylogenetic signal to discriminate closely related *Polygala* species and to recover species-level relationships consistent with plastome-scale analyses. By concatenating nine chloroplast regions identified through nucleotide diversity analysis, we were able to reconstruct a species-level phylogeny that closely mirrored the topology obtained from complete chloroplast gene datasets. Our results indicate that these less commonly used regions may substantially contribute to phylogenetic resolution when combined in a multi-locus framework. Taken together, the integration of nucleotide diversity screening, targeted marker selection, and concatenated phylogenetic analysis offers a robust framework for DNA barcoding studies in *Polygala*. In this study, we tested their abilities of species identification but the other barcoding standards such as the universality of these markers, PCR success and sequence quality will be needed to test for rapid and accurate species identification in future.

In *P. vulgaris*, most of the nucleotide diversity values in the LSC, IR and SSC regions were greater than the mean values but IR regions had only two hot spot regions. Jeong et al. (2025) analyzed the nucleotide diversity in three *Polygala* chloroplast genomes and found similar results that the IR regions were more conserved than the LSC and SSC regions. Moreover, the Pi reported as 0.0096, is not in agreement with our finding. The overall Pi was found as 0.10970, and nine hypervariable gene regions were detected with a Pi value above 0.22 in this study.

The tetraploid species *P. vulgaris* is found in European countries, including Türkiye, and has important secondary metabolites for medicine (Çalış et al. 2025). Despite its pharmacological efficacy and cytological knowledge, the species has morphological variations, and it is necessary to clarify its phylogenetic position. The use of the cp genome has become increasingly common in the phylogeny of angiosperms (Hollingsworth et al. 2011; Xiong et al. 2023; Zhai et al. 2019) and offers advantages such as uniparental inheritance, small size, well-conserved structure, and moderate nucleotide variation (Mower and Vickrey 2018; Palmer 1985). Previous phylogenetic studies of *Polygala* have been based on several molecular markers sequenced via the Sanger method (Çeçen et al. 2023; Eriksen 1993; Lyskov et al. 2019; Martinez et al. 2022; Pastore et al. 2019; Persson 2001), whereas Lee et al. (2020), Ma et al. (2021), Wang (2023; 2025), Jeong et al. (2025), and our study produced relatively high phylogenetic resolution by utilizing plastome data. In this study, the phylogenomic tree yielded reliable results, confirming that *P. vulgaris* is a distinct species closely related to the European taxon *P. amerella*. Andrew J. Lack (1995) searched for putative hybrids between diploid *P. calcarea* and *P. vulgaris* and reported that both species hybridize with tetraploid *P. vulgaris* on the basis of isozyme enzymes. The phylogenetic tree inferred from the BI and ML analyses revealed that *P. vulgaris* separated from *P. amerella* and *P. calcarea,* a finding that corroborated the previous studies of Pastore et al. (2019). In the phylogenetic tree, *P. vulgaris* and *P. amerella* were clustered together with strong support values. However, Pastore et al. (2019) reported that *P. amerella* is more closely related to *P. calcarea* than to *P. vulgaris* on the basis of *trnL-F*, *rbcL*, and *trnK-matK* and the nuclear ribosomal ITS. Overall, the current taxon sampling methods are still limited, and the cp genome data of other European taxa should be evaluated by combining evidence from the chromosome evolution of *P. vulgaris*.

In addition, an important evolutionary aspect of the present study is the comparison between the tetraploid cytotype of *P. vulgaris* and the diploid cytotype of *P. tenuifolia* (Meng et al. 2023). Although polyploidization primarily affects the nuclear genome, cytonuclear interactions and genome dosage effects may indirectly influence plastid genome evolution (Soltis et al. 2015; Peer et al. 2017; Wicke et al. 2011). In this context, the largely conserved plastome structure observed between the tetraploid *P. vulgaris* and the diploid *P. tenuifolia* suggests a high level of chloroplast genome stability across different ploidy levels in *Polygala*. This conservation is reflected in multiple plastome features, including overall genome size and quadripartite organization, highly similar gene content and gene order, comparable distributions of repeat sequences and simple sequence repeats (SSRs), and the absence of extensive gene loss or pseudogenization. These patterns are consistent with previous plastome-based studies showing that chloroplast genomes generally remain structurally stable despite changes in nuclear ploidy, owing to their uniparental inheritance and independent evolutionary dynamics (Palmer 1985; Wicke et al. 2011; Soltis et al. 2015).

The analysis of relative synonymous codon usage (RSCU) in the chloroplast genome of *P. vulgaris* revealed a codon usage pattern largely consistent with that observed across other *Polygala* species. Like many angiosperm chloroplast genomes, codons ending with A or U are strongly favored, reflecting a general bias toward A/U at the third codon position (Dai et al. 2023; Wang et al. 2020; Zou et al. 2025). The universal initiation codon AUG (Met) likewise displayed high relative usage. Although the overall codon usage patterns were highly conserved among *Polygala* species, subtle interspecific differences were detected, particularly within the sets of synonymous codons for leucine and serine. These minor deviations may reflect lineage-specific evolutionary pressures, such as mutation bias, translational selection, or genetic drift, that can shape codon preferences within chloroplast genomes (Chakraborty et al. 2020; Xiao et al. 2024; Yang et al. 2024). Importantly, the codon usage bias observed in *P. vulgaris* did not diverge markedly from that of its congeners, supporting the notion that codon usage in *Polygala* chloroplast genomes is relatively stable and conserved. Taken together, these results highlight that the chloroplast genomes of *Polygala* retain the characteristic A/U bias typical of higher plants while also exhibiting species-specific codon usage signatures that may provide insights into their evolution.

The distribution of repeat sequences showed considerable variation among the *Polygala* species analyzed. Forward repeats were generally the most abundant repeat type, followed by palindromic and reverse repeats, which is consistent with observations in other angiosperm plastomes (Guo et al. 2022; Tseng et al. 2025; Wu et al. 2021a). In *P. vulgaris,* the total number of repeats was moderate compared with that in the other species, with relatively balanced proportions of forward and palindromic repeats. The presence of a relatively high proportion of certain repeat classes may suggest potential hotspots for genome rearrangements or play a role in maintaining plastome stability and these studies have highlighted the role of repeats in plastome evolution, including structural variation and recombination events (Asaf et al. 2020; Wu et al. 2021a; Xu et al. 2023). Our findings for *P. vulgaris* provide further evidence that repeat content may contribute to lineage-specific patterns of plastid genome evolution within *Polygala*. The comparative SSR analysis revealed that mononucleotide repeats, particularly A/T motifs, dominated all *Polygala* plastomes in this study. This strong A/T bias is a common feature of angiosperm chloroplast genomes and has been widely attributed to the AT-rich nature of plastid DNA and replication mechanisms (Palmer 1985; Wicke et al. 2011; Daniell et al. 2016). Although the general SSR composition was highly conserved across species, moderate interspecific variation in total SSR number and repeat class distribution was detected. Such variation may reflect lineage-specific mutation dynamics and population-level divergence, and it provides a useful genomic resource for developing polymorphic chloroplast SSR markers. These markers have proven valuable tools for studies of species identification, phylogeography, and genetic diversity in closely related taxa.

Importantly, the limited presence of higher-order SSR motifs (tri- and tetranucleotide repeats) across *Polygala* plastomes suggests a relatively stable plastome architecture. The single tetranucleotide SSR detected in *P. tenuifolia* further supports the notion that long repeat motifs are rare but may represent lineage-specific evolutionary signatures. Together, these findings highlight the potential of plastid SSRs as complementary molecular resources for future taxonomic, population genetic, and barcoding studies within *Polygalaceae*.

## Conclusion

The comparative analysis of nucleotide diversity, repeat sequences, genome synteny, and phylogenetic relationships provides complementary insights into the evolutionary dynamics of *Polygala* chloroplast genomes. Our repeat analysis revealed that *P. vulgaris* has a distinctive distribution of forward, reverse, and palindromic repeats. Such repeats, particularly forward and palindromic repeats, are known to facilitate recombination and may underlie subtle structural polymorphisms, especially near IR boundaries and intergenic spacers. Despite this variability, the synteny comparison demonstrated that the overall gene order across *Polygala* species remains highly conserved, indicating a structurally stable plastome with no evidence of large-scale rearrangements. This structural conservation is consistent with the phylogenetic reconstruction, which strongly supported *P. vulgaris* as a sister to *P. amerella*. Nine hypervariable regions were detected and their discrimination ability were tested based on concatenated DNA dataset.

This research enhances our knowledge of chloroplast genome evolution in *Polygala*, ensuring that a plastome resource contributes to understanding evolution of organelle genomes, phylogenomics and DNA barcoding.

## Data Availability

The raw reads used in this study were deposited in the Sequence Read Archive (SRA) NCBI accession number PRJNA1139969.
